# Supplementary material for: Intimate partner violence and malnutrition among women of reproductive age in Western Africa: A geostatistical analysis
Source: PLOS Glob Public Health. 2023 Nov 8;3(11):e0002354. doi: 10.1371/journal.pgph.0002354 (PMC10631639; doi:10.1371/journal.pgph.0002354)
Supplement: S1 Text — (DOCX) [file pgph.0002354.s001.docx]

Table A1: Questions used by Demographic and Health Survey to elicit information on each type of intimate partner violence

| **Physical violence** | **Sexual violence** | **Emotional violence** |
| --- | --- | --- |
| - Pushing or shaking you or throwing something at you - Slapping you - Twisting your arm or pulling your hair - Punching you with his fist or with something that could hurt you - Kicking you, dragging you or beat you up - Threaten or attack you with a knife, gun, or any other weapon - Trying to choke you or burn you on purpose | - Physically forcing you to have sexual intercourse with him even when you did not want to - Physically forcing you to perform any other sexual acts you did not want to - Forcing you with threats or in any other way to perform sexual acts you did not want to | - Saying or doing something to humiliate you in front of others - Threaten to hurt or harm you or someone close to you - Insulting you or making you feel bad about yourself |
