# Supplementary material for: Intimate partner violence and malnutrition among women of reproductive age in Western Africa: A geostatistical analysis
Source: PLOS Glob Public Health. 2023 Nov 8;3(11):e0002354. doi: 10.1371/journal.pgph.0002354 (PMC10631639; doi:10.1371/journal.pgph.0002354)
Supplement: Attachment — Submitted filename: Reviewers comment on Intimate partner violence and malnutrition among women of reproductive age in Western Africa-A geostatistical analysis.docx [file pgph.0002354.s002.docx]

**Reviewer’s comments**

**Abstract section**

1. The abstract is too short and does not contain all relevant information that it should contain as per the standard of PLOS ONE. **Therefore, this issue must be addressed.**

**Background section**

Well done but the following comment should be considered

1. The introduction is too long because it contains many ideas that are not directly related to the research topic. So, it should focus on the study topic.
2. The research objective and the research contribution to the world are not explained.

**Method section**

Well done but the following comment should be considered

1. In this section the software used for data analysis is not mentioned.
2. The statistical description method is not indicated
3. The data source section is unnecessarily too long as it contains many irrelevant information such as how DHIS Data were collected and analyzed. So, shorten it.
4. Justification should be given why the authors prefer to use bi-probit and Bayesian approach.

**Result section**

1. In this section the magnitude or coefficients of the variable s that were compared along with their respective confidence or correlation interval are not shown.

2. The section is congested by presenting all results in one paragraph that makes the result section boring. Thus, to make it attractive the result section should be revisited and each table and figure should be presented after narration.

3. Furthermore, this section like the introduction section is too long. So the section should focus on

**Discussion section**

This section should be revisited by looking at the following comments

1. It is too long so it should be shortened by focusing on the important points as I commented on result section.

2. The interpretations of results are not clear for reader so that the authors should have to interpret results and compare with the relevant literatures in a way it is understandable.

3. The interpretation should lead to the conclusion and recommendations. Also, it is a must to make sure result answer the research questions and achieve the research objectives.

**Conclusion Section**

The conclusion section should be revisited again as per the comments given above and in the research word document.
