# Supplementary material for: Intimate partner violence and malnutrition among women of reproductive age in Western Africa: A geostatistical analysis
Source: PLOS Glob Public Health. 2023 Nov 8;3(11):e0002354. doi: 10.1371/journal.pgph.0002354 (PMC10631639; doi:10.1371/journal.pgph.0002354)
Supplement: Attachment — Submitted filename: RESPONSE TO COMMENTS clean.docx [file pgph.0002354.s003.docx]

# **RESPONSE TO COMMENTS BY REVIEWERS**

**Manuscript Title:** Intimate partner violence and malnutrition among women of reproductive age in Western Africa: A geostatistical analysis

Manuscript Number:

**Author(s) response to comments by reviewers**

| **Section of manuscript** | **Reviewer Comment** | **Author(s) Response** |
| --- | --- | --- |
| **REVIEWER #1** | | |
| **Abstract** | 1. Thank you for inviting to review this article but my concern is it is not following scientific background to write an article which will be published in reputable journal like PLOS Global Public Health. Abstract of a manuscript which includes at least, Background, objective, method, result, and conclusion. But this is not shows what it follows, and the abstract of this manuscript is not explanatory and gives sound for any one to grasp the overall the manuscript in its abstract. | We would like to thank the reviewer for this comment. The abstract has been revised to include all necessary sections though still in a blocked form as the journal requires it. |
| **Introduction** | 2. Online “59” Introduction part next to reference 7, there is Harvard style reference. Why it is why not be consistent and be Vancouver style like others? | All references have been formatted using the Vancouver style.  Thank you. |
| **Results** | 3. it is better to put statistical measures result which have an association on abstract result and discussion, because it is not possible to know which variable affects positively and negatively the association of intimate partner violence and malnutrition | Statistical measures have been included in the abstract, results and discussions where applicable.  Thank you. |
| **REVIEWER #2** | | |
| **Abstract section** | 1. The abstract is too short and does not contain all relevant information that it should contain as per the standard of PLOS ONE. **Therefore, this issue must be addressed.** | Thank you for the observation. The abstract has been structured in line as per PLOS Global Public Health standard and the relevant information have been included accordingly. |
| **Background section** | Well done but the following comment should be considered: | We would like to thank the reviewer for these comments. The manuscript has been revised recommendations of the reviewer as follows: |
|  | 1. The introduction is too long because it contains many ideas that are not directly related to the research topic. So, it should focus on the study topic. | The introduction section has been significantly shortened. For example, the statistics on the different types of IPV and their prevalence across different countries have been taken out of the introduction.  Also, the paragraphs on the mechanism of how IPV contributes to malnutrition among women and children have been taken out of the write up. Please see pages 2 and 3 of the revised manuscript. |
|  | 2. The research objective and the research contribution to the world are not explained. | The research objective and the research contribution have been explained in the revised manuscript. The revision has been highlighted in red on page 3 of the revised manuscript. |
| **Method section** | Well done but the following comment should be considered | We would like to thank the reviewer for these comments. The comments have been addressed as follows: |
|  | 1. In this section the software used for data analysis is not mentioned. | The software used for the analysis is BayesX. We have now mentioned the version as version 3.0.2. Please see the methods of section of the revised manuscript. Thank you. |
|  | 2.The statistical description method is not indicated | We would like to thank the reviewer for this observation. A detailed description of the statistical method, including the types of models, the reasons for the choice of the models and the equation for model specification have been provided. Please see the methods of analysis section of the revised manuscript for the revision. |
|  | 3. The data source section is unnecessarily too long as it contains many irrelevant information such as how DHIS Data were collected and analyzed. So, shorten it. | The data source section of the methods has been shortened, moving information on the questions used to create the intimate partner violence indicators to the appendix. Thank you. |
|  | 4. Justification should be given why the authors prefer to use bi-probit and Bayesian approach. | We have provided the basis for choosing a bi-probit model and a Bayesian approach for inference.  For the choice of the model, we stated that “The choice of this model is based on the assumption that the two outcome variables of interest: underweight and IPV are both binary indicators which can be highly correlated as earlier argued.”  For using a Bayesian approach, we stated “Because of the complexity of the model parameters that involve covariates of different types, a Bayesian approach is a suitable approach for parameter estimation.” |
| **Result section** | 1. In this section the magnitude or coefficients of the variables that were compared along with their respective confidence or correlation interval are not shown. | We appreciate the reviewer for this concern. However, based on your other comments that indicate that the result section appears long, we feel, adding the coefficients in describing the results in this section will further increase the length. We therefore refer to them from the Tables where they are already presented. |
|  | 2.The section is congested by presenting all results in one paragraph that makes the result section boring. Thus, to make it attractive the result section should be revisited and each table and figure should be presented after narration. | We appreciate this concern and have reviewed the results section, breaking into paragraphs and subsections for clarity. Based on the instruction of the journal, we presented the results at the end of the manuscript but hope that they will be placed close to where they have been described in the final version. |
|  | 3. Furthermore, this section like the introduction section is too long. So the section should focus on | We have edited the section, cutting down on unnecessary presentations so that the section is clearly presented. Thank you. |
| **Discussion section** | This section should be revisited by looking at the following comments | We would like to thank the reviewer for these comments. The comments have been addressed as follows: |
|  | 1. It is too long so it should be shortened by focusing on the important points as I commented on result section. | The discussion section has been shortened. The points of discussion have been limited to salient findings in the results section. Thank you. |
|  | 2. The interpretations of results are not clear for reader so that the authors should have to interpret results and compare with the relevant literatures in a way it is understandable. | The interpretations of the results and the associated discussion have been made clearer and more concise for each of understanding. Thank you. |
|  | 3.The interpretation should lead to the conclusion and recommendations. Also, it is a must to make sure result answer the research questions and achieve the research objectives. | The conclusion has been revised to reflect the research questions and objectives. The conclusion summarises the spatial clustering in IPV and malnutrition among women of reproductive age in West Africa and highlights the factors associated with IPV and malnutrition as identified in this study. |
| **Conclusion Section** | The conclusion section should be revisited again as per the comments given above and in the research word document. | The conclusion has been revisited. The revised conclusion summarises the spatial clustering in IPV and malnutrition among women of reproductive age in West Africa and highlights the factors associated with IPV and malnutrition as identified in this study. |
