# Supplementary material for: Intimate partner violence and malnutrition among women of reproductive age in Western Africa: A geostatistical analysis
Source: PLOS Glob Public Health. 2023 Nov 8;3(11):e0002354. doi: 10.1371/journal.pgph.0002354 (PMC10631639; doi:10.1371/journal.pgph.0002354)
Supplement: Attachment — Submitted filename: Reviewers comment on Intimate partner violence and malnutrition among women of reproductive age in Western Africa-A geostatistical analysis09232023.docx [file pgph.0002354.s004.docx]

**Reviewer’s comments**

**Abstract section**

1. This section is well addressed as per the comment but it does not indicate the conclusion and recommendation

**Background section**

Well done but the following comment should be considered

1. The research objective and the research contribution to the world is not explained.

**Method section**

Well done but the following comment should be considered

1. In this section the software used for data analysis is not mentioned.
2. Justification should be given why the authors prefer to use bi-probit and Bayesian approach.

**Result section**

1. It is well addressed

**Discussion section**

This section should be revisited by looking at the following comments

1. It is well addressed

**Conclusion Section**

It is well addressed
