# Supplementary material for: Intimate partner violence and malnutrition among women of reproductive age in Western Africa: A geostatistical analysis
Source: PLOS Glob Public Health. 2023 Nov 8;3(11):e0002354. doi: 10.1371/journal.pgph.0002354 (PMC10631639; doi:10.1371/journal.pgph.0002354)
Supplement: Attachment — Submitted filename: Author Response to Comments_ROUND 2 REVISION.docx [file pgph.0002354.s005.docx]

**RESPONSE TO COMMENTS BY REVIEWERS**

**Manuscript Title:** Intimate partner violence and malnutrition among women of reproductive age in Western Africa: A geostatistical analysis

Manuscript Number:

**Author(s) response to comments by reviewers**

| **Section of manuscript** | **Reviewer Comment** | **Author(s) Response** |
| --- | --- | --- |
| **COMMENTS BY EDITOR** | | |
| **Section of manuscript** | **Comment** | **Response by Authors** |
| **Journal Requirements** | 1. Please review your reference list to ensure that it is complete and correct. If you have cited papers that have been retracted, please include the rationale for doing so in the manuscript text or remove these references and replace them with relevant current references. Any changes to the reference list should be mentioned in the rebuttal letter that accompanies your revised manuscript. If you need to cite a retracted article, indicate the article’s retracted status in the References list and also include a citation and full reference for the retraction notice. | We have reviewed the references to ensure that only those cited were listed in the reference section, and none of them has been retracted. |
|  | 2. Please provide separate figure files in .tif or .eps format only and remove any figures embedded in your manuscript file. Please also ensure all files are under our size limit of 10MB. | The Figures have been removed from the manuscript and provided separately as .tif. |
|  | 3. We have noticed that you have uploaded Supporting Information files, but you have not included a list of legends. Please add a full list of legends for your Supporting Information files after the references list. | We have provided the legend for the Supporting information immediately after the reference list as requested. Thank you. |
| **REVIEWER’S COMMENTS** | | |
| **Abstract section** | 1. This section is well addressed as per the comment but it does not indicate the conclusion and recommendation | 1. We would like to thank the reviewer for this comment. The entire abstract has been revised. The conclusions and recommendations have been included at the end of the abstract in the revised manuscript. |
| **Background section** | Well done but the following comment should be considered   1. The research objective and the research contribution to the world is not explained. | 1. The research objective and contribution to knowledge has been incorporated in the last paragraph of the introduction. Thank you. |
| **Method section** | Well done but the following comment should be considered  1. In this section the software used for data analysis is not mentioned.  2. Justification should be given why the authors prefer to use bi-probit and Bayesian approach. | Thank you so much for the comments. We have emphasized, at the beginning of the method section, the basis for choosing a bi-probit model. The reason for choosing a Bayesian approach has also been stated in lines 160-162. Bayesian method allow for sampling from the posterior distributions that are often intractable because of the complex nature of the model, such as ours that involves variables of different types.  We stated the software used for the study in lines 182-183 thus: BayesX version 3.0.2 – a freely distributed software package for Bayesian inference in structured additive regression models. |
| **Result section** | 1. It is well addressed | Thank you. |
| **Discussion section** | This section should be revisited by looking at the following comments   1. It is well addressed | Thank you. |
| **Conclusion Section** | 1. It is well addressed | Thank you. |
